# Supplementary material for: A self-driving laboratory advances the Pareto front for material properties
Source: Nat Commun. 2022 Feb 22;13:995. doi: 10.1038/s41467-022-28580-6 (PMC8863835; doi:10.1038/s41467-022-28580-6)
Supplement: Supplementary file 1 — Supplementary information [file 41467_2022_28580_MOESM1_ESM.pdf]

Supplementary Information

A self-driving laboratory advances the Pareto front for material properties

Benjamin P. MacLeod[1,2,*], Fraser G. L. Parlane[1,2,*], Connor C. Rupnow[1,2,3], Kevan E. Dettelbach[1], Michael S. Elliott[1], Thomas D. Morrissey[1,2], Ted H. Haley[1], Oleksii Proskurin[1], Michael B. Rooney[1], Nina Taherimakhsousi[1], David J. Dvorak[2], Hsi N. Chiu[1], Christopher E. B. Waizenegger[1], Karry Ocean[1], Mehrdad Mokhtari[1]& Curtis P. Berlinguette[1,2,3,4,†]

[1]Department of Chemistry, The University of British Columbia, 2036 Main Mall, Vancouver, BC V6T 1Z1, Canada.
[2]Stewart Blusson Quantum Matter Institute, The University of British Columbia, 2355 East Mall, Vancouver, BC V6T 1Z4, Canada.
[3]Department of Chemical and Biological Engineering, The University of British Columbia, 2360 East Mall, Vancouver, BC V6T 1Z3, Canada.
[4]Canadian Institute for Advanced Research (CIFAR), MaRS Centre, 661 University Avenue Suite 505, Toronto, ON M5G 1M1, Canada.

*These authors contributed equally to this work.
[†]Corresponding author. Email: cberling@chem.ubc.ca

Supplementary Tables

Supplementary Table 1 | Initialization points used for the optimizations

sample	fuel blend (x)	anneal temperature (T, °C)	fuel to oxidizer ratio (ϕ)	concentration (C, mg mL^{-1})	conductivities across campaigns 1-4 mean (S m^{-1})	conductivities across campaigns 1-4 std. dev. (S m^{-1})
1	0.5	220	2.0	0.006	21	12
2	0.7	260	0.5	0.012	31	16
3	1.0	200	2.0	0.012	8	2
4	0	220	0.5	0.006	68	8
5	0.1	260	1.0	0.012	37	11
6	0.7	200	0.5	0.012	1	1
7	0.9	220	1.0	0.006	67	7
8	1.0	260	2.0	0.012	17	10

Supplementary Table 2 | Recipes from the Pareto front chosen for spray coating

recipe	fuel blend (x)	anneal temperature (T, °C)	fuel-to-oxidizer ratio (ϕ)	concentration (C, mg mL^{-1})
1	0.78	191	1.56	0.0066
2	0.85	200	0.32	0.0065
3	0.90	226	1.17	0.0060

Supplementary Table 3 | Spray-coated sample characterization results

spray-coated sample recipe	thickness, mean (nm)	thickness, std. dev. (nm)	thickness, std dev. / mean (%)	conductivity, mean (S m^{-1})	conductivity, std. dev. (S m^{-1})	conductivity, std. dev. / mean (%)
1	49.2	2.2	4.5	3.3×10^4	6×10^3	17.4
	50.5	2.4	4.8	1.25×10^5	1.3×10^4	11.0
	50.6	2.0	4.0	1.7×10^5	2×10^4	12.1
2	61.1	1.4	2.3	3.89×10^5	1.1×10^4	2.8
	59.0	1.6	2.7	4.0×10^5	3×10^4	8.0
	59.7	2.1	3.5	3.97×10^5	8×10^3	2.0
3	48.3	1.4	2.9	1.88×10^6	8×10^4	4.4
	54.2	0.9	1.7	1.95×10^6	3×10^4	1.6
	51.9	1.6	3.1	2.05×10^6	6×10^4	2.8

Supplementary Table 4 | Leave-One-Out Cross-Validation analysis of simulation models

model	RMSE (S m^{-1})	NRMSE (range; %)	MAE (S m^{-1})	r^2	bias (S m^{-1})
**Single campaigns**					
campaign 1 (2020-12-18_17-38-40)	10.6	10.4	7.699	0.836	0.092
campaign 2 (2020-12-23_17-06-50)	13.8	15.5	10.961	0.693	-0.016
campaign 3 (2021-01-04_08-37-39)	10.7	11.2	7.671	0.828	0.287
campaign 4 (2021-01-12_16-26-56)	11.6	9.2	7.238	0.871	0.336
**Combined campaigns**					

all campaigns (no noise)	12.4	9.8	9.116	0.800	-0.123
all campaigns (with noise)	16.3	12.8	12.064	0.659	1.820

Supplementary Methods

Calculation of the fuel to oxidizer ratio for the combustion synthesis reaction

An idealized form for the combustion synthesis reaction studied here is:

$$\mathrm{Pd(NO_3)_2} + \frac{\varphi}{\frac{10}{9}(1-x)+\frac{10}{24}x}(x\cdot\mathrm{acac}+(1-x\cdot\mathrm{gly})+(\varphi-1)\mathrm{O_2} \rightarrow \mathrm{Pd}+\mathrm{gases}$$

This assumes that the available oxidizers and fuels react fully with each other, and with additional atmospheric oxygen as needed to yield pure, metallic palladium. This idealized reaction is based on Jain's method[1] which calculates an overall fuel-to-oxidizer ratio on the basis of the oxidizing or reducing valence of each species involved in a combustion reaction. The reducing valences used are +4 for carbon, +1 for hydrogen, 0 for nitrogen, -2 for oxygen and +*v* for a metal forming a compound in which the metal has formal charge *v* (e.g. +2 for Pd).

Transformations from normalized optimizer variables to dimensional variables used by the robotics

As shown in Fig. 2, the optimizations performed here were cast in terms of normalized variables:

- The fuel to oxidizer ratio, φ (dimensionless)
- The fuel blend, *x* (dimensionless)
- The total precursor concentration, *C* (mg/mL)
- The annealing temperature, *T* (°C)

The values for these variables were chosen by the qEHVI optimization algorithm before each experiment. While the annealing temperature *T* chosen by the algorithm could be passed directly to the robotic hardware, the other variables (φ, *x*, and *C*) required transformation into quantities suitable for execution by the robot. Specifically, a transformation was applied to these quantities to determine the required volumes of the $Pd(NO_3)_2$, glycine, and acetylacetone stock solutions and water diluent required to form the precursor ink for each experiment. The transformations required that the total volume of ink required ink be specified (200 µL for the present experiments) and are expressed in terms of the following quantities:

- The normalized variables introduced above (φ, *x*, and *C*)
- The total volume of ink to be mixed ($V_{\mathrm{ink}} = 200\mu L$)
- The required volume of $Pd(NO_3)_2$ solution (V_{Pd})
- The required volume of glycine solution solution (V_{gly})
- The required volume of acetylacetone solution (V_{acac})
- The required volume of water used as diluent (V_{diluent})
- The reducing valences and molar masses of each compound involved in the combustion, as well as the concentrations of the associated stock solutions:

Compound	reducing valence	molar mass (g mol^{-1})	stock solution concentration (mg mL^{-1})
$Pd(NO_3)_2$	R_{Pd} = -10	MM_{Pd} = 230.43	C_{Pd} = C_{stocks} = 12
glycine	R_{gly} = 9	MM_{Gly} = 75.07	C_{Gly} = C_{stocks} = 12
acetylacetone	R_{acac} = 24	MM_{Acac} = 100.13	C_{Acac} = C_{stocks} = 12

The volumes of chemicals used are found by solving the following equations numerically:

$$\frac{V_{\text{gly}}}{V_{\text{Pd}}} = \phi\,(1-x)\left(\frac{R_{\text{Pd}}}{R_{\text{gly}}}\right)\left(\frac{M_{\text{gly}}}{M_{\text{Pd}}}\right)$$

$$\frac{V_{\text{acac}}}{V_{\text{Pd}}} = \phi\,(x)\left(\frac{R_{\text{Pd}}}{R_{\text{acac}}}\right)\left(\frac{M_{\text{acac}}}{M_{\text{Pd}}}\right)$$

$$V_{\text{ink}} = V_{\text{Pd}} + V_{\text{gly}} + V_{\text{acac}} + V_{\text{diluent}}$$

$$\frac{V_{\text{ink}} - V_{\text{diluent}}}{V_{\text{ink}}} = \frac{C}{C_{\text{stocks}}}$$

Manual screening experiments

We qualitatively compared the decomposition temperatures of palladium combustion synthesis precursors using manual screening experiments. In these experiments, precursors containing glycine, urea, or acetylacetone as the fuel were drop cast onto glass substrates and allowed to dry in air. The dried precursors were then placed on a hotplate preheated to a specified temperature and observed by eye. Metallic films were never obtained for hotplate temperatures below 180 °C. The precursors containing glycine or acetylacetone exhibited a change in appearance earlier than those containing urea and were found to yield conductive films after annealing on a hotplate set to 350 °C. Conductive films were obtained from the urea-containing precursors only upon further heating.

Supplementary Figures

All figures containing numerical data were created in Python using the matplotlib library.

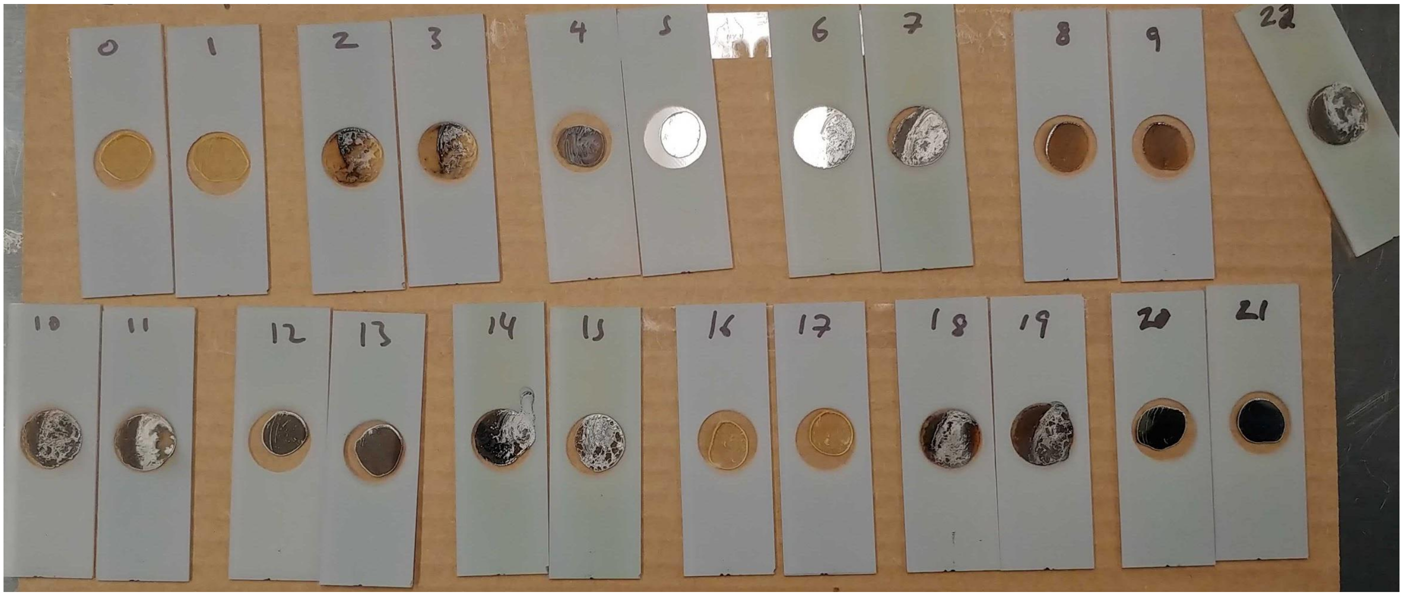

Supplementary Figure 1 | Photographs of typical drop casted films created by the robotic laboratory. The substrates are 3” × 1” glass microscope slides. A grey spray paint which is poorly wet by the precursors was used to define an 18-mm diameter circular well at the center of each slide. The precursors are dispensed into this well.

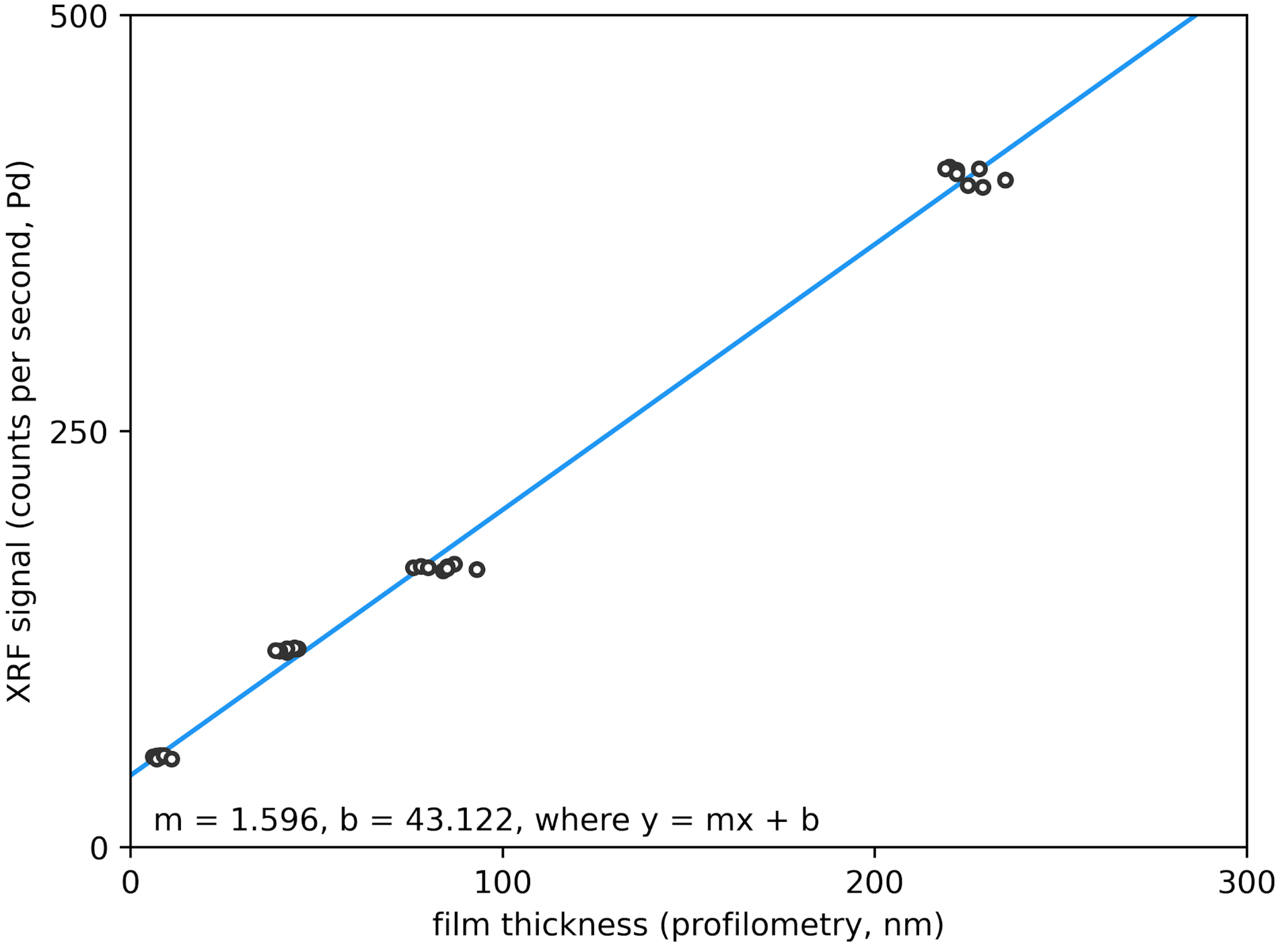

Supplementary Figure 2 | Characterization of sputtered palladium films used for XRF calibration. Films of nominal thickness 10 nm, 50 nm, 100 nm, and 250 nm were deposited by sputtering and then characterized using XRF and profilometry. A linear relationship was observed between film thickness and XRF intensity.

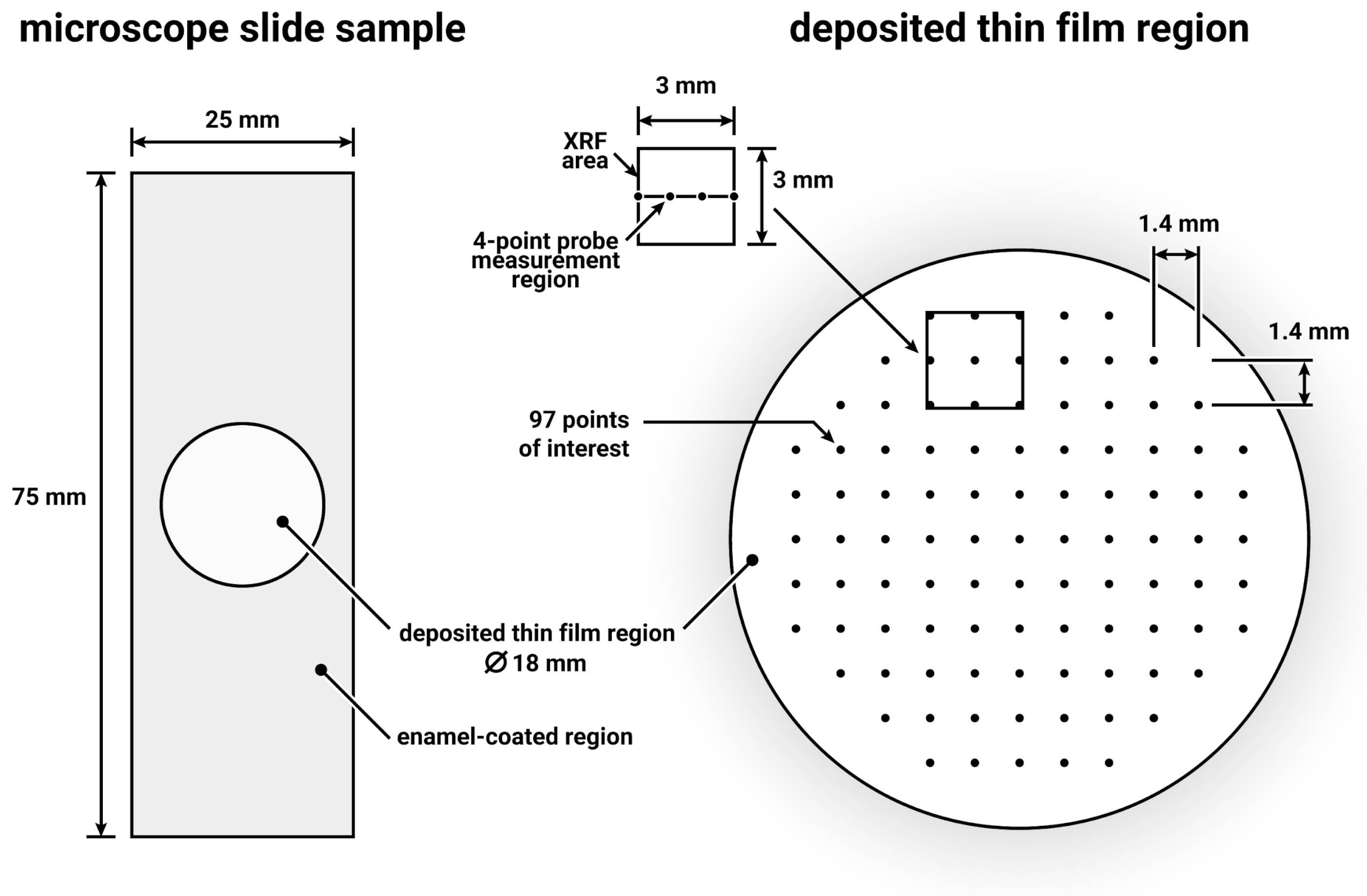

Supplementary Figure 3 | Layout of the microscope slide sample and measurement regions. Each microscope slide has an enamel coating that masks out a region on the slide with a diameter of 18 mm. The palladium thin film is deposited in this region. The conductance is measured with a 4-point probe at each point of interest. The XRF signal for palladium is measured over a 3 mm by 3 mm region centered on each point of interest.

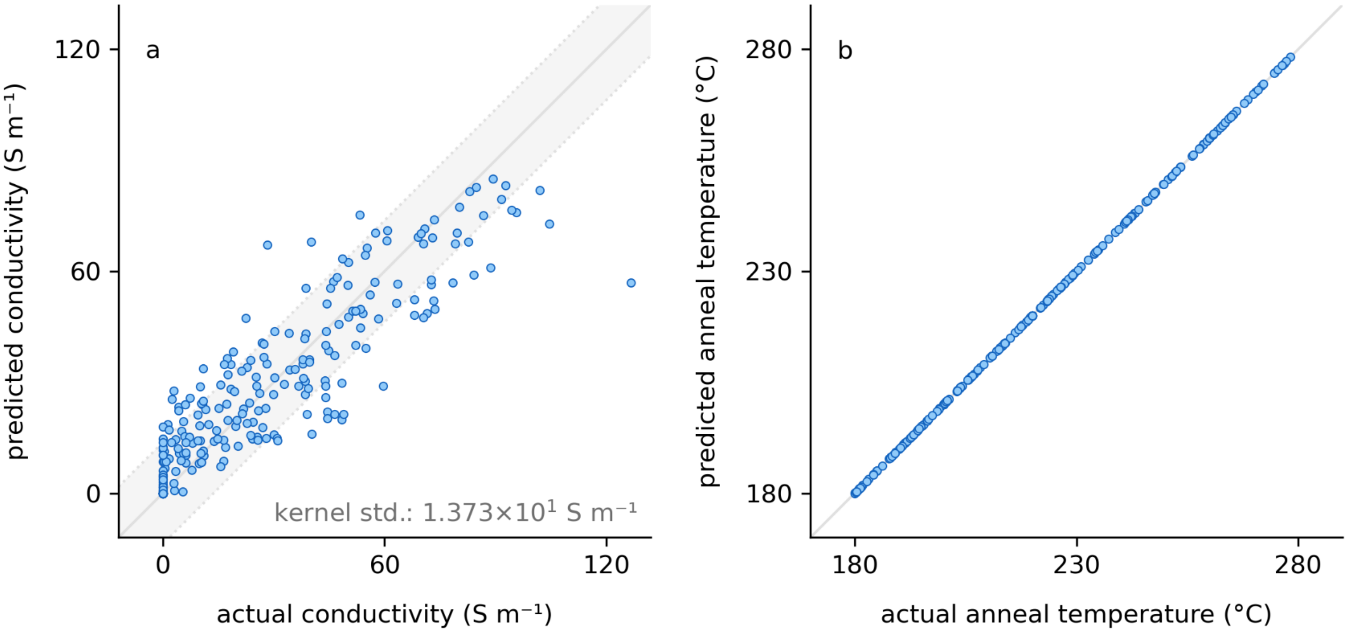

Supplementary Figure 4 | Leave-One-Out Cross-Validation (LOOCV) analysis of the noise-free model of the experimental response surfaces for (a) conductivity and (b) temperature. The model was created by training a Gaussian process model on all the combined data from all four optimization campaigns. (See methods.) The amplitude of the experimental noise in the conductivity training data was estimated using a white noise kernel in the Gaussian process regression model. This noise estimate is plotted as a grey band spanning ±1 standard deviation.

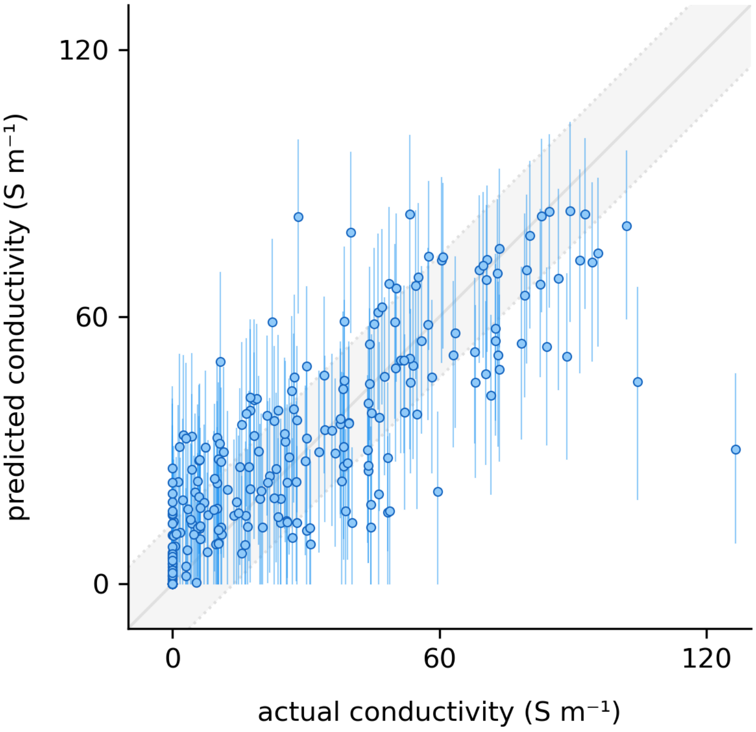

Supplementary Figure 5 | Leave-One-Out Cross-Validation (LOOCV) analysis of the noisy model of the experimental response surface. The median (blue dots) and interquartile range (blue bars) of the noise model is reported by sampling the noise model 1×10^6 times at each point in the LOOCV analysis. The amplitude of the experimental noise in the conductivity training data was estimated using a white noise kernel in the Gaussian process regression model. This noise estimate is plotted as a grey band spanning ±1 standard deviation.

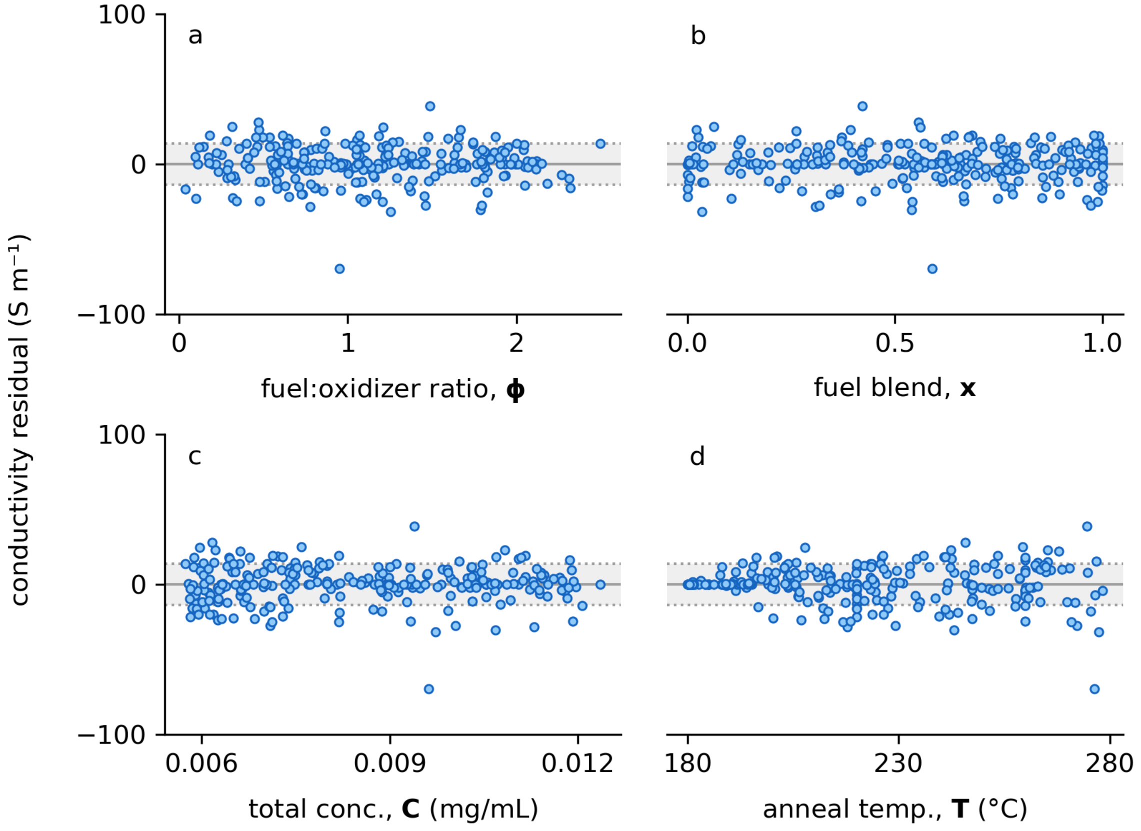

Supplementary Figure 6 | Model residuals as a function of model inputs. The conductivity residuals from the Leave-One-Out Cross-Validation (LOOCV) analysis are plotted as a function of the input parameters: (**a**) fuel:oxidizer ratio, (**b**) fuel blend, (**c**) total concentration and (**d**) annealing temperature. These plots reveal that the model uncertainty has little structure across the input space. The amplitude of the experimental noise in the conductivity training data was estimated using a white noise kernel in the Gaussian process regression model. This noise estimate is plotted as a grey band spanning ±1 standard deviation.

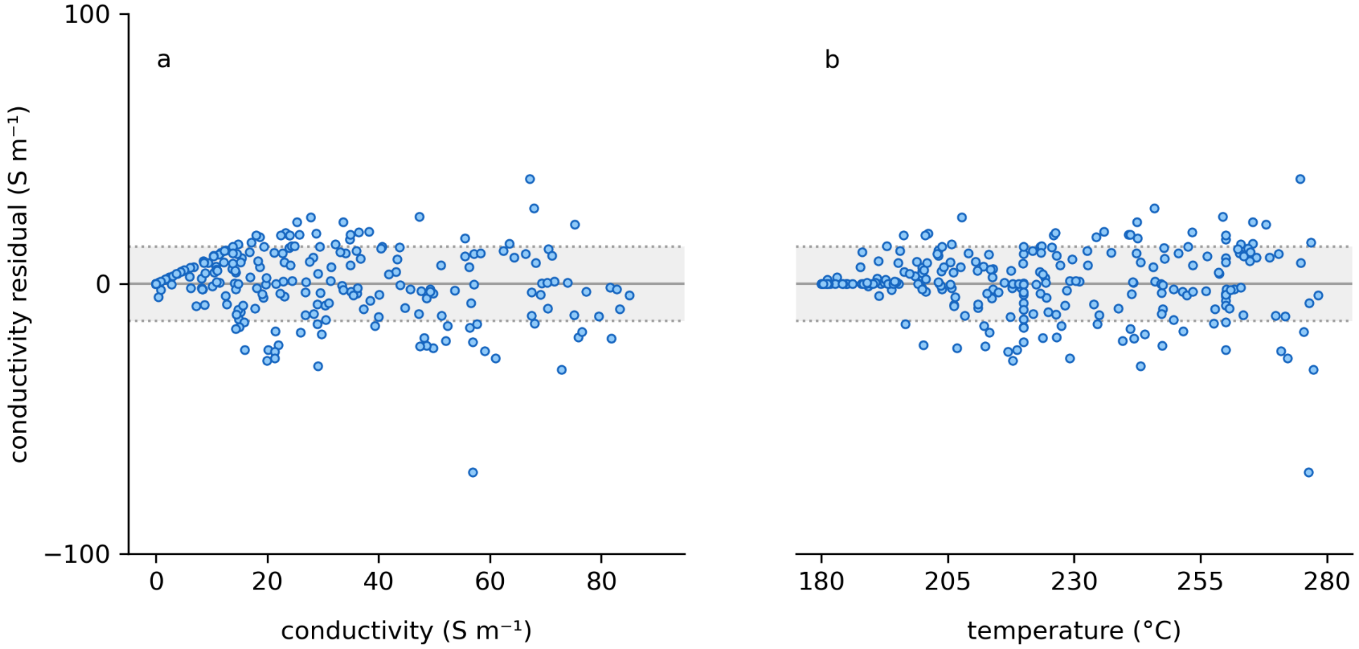

Supplementary Figure 7 | Model residuals as a function of model outputs. The conductivity residuals from the Leave-One-Out Cross-Validation (LOOCV) analysis are plotted as a function of the model outputs: (**a**) conductivity and (**b**) annealing temperature. Note that the structure seen in panel a from 0 to 20 S m^{-1} is a result of predicted conductivities that are negative being clipped to 0 S m^{-1}. The amplitude of the experimental noise in the conductivity training data was estimated using a white noise kernel in the Gaussian process regression model. This noise estimate is plotted as a grey band spanning ±1 standard deviation.

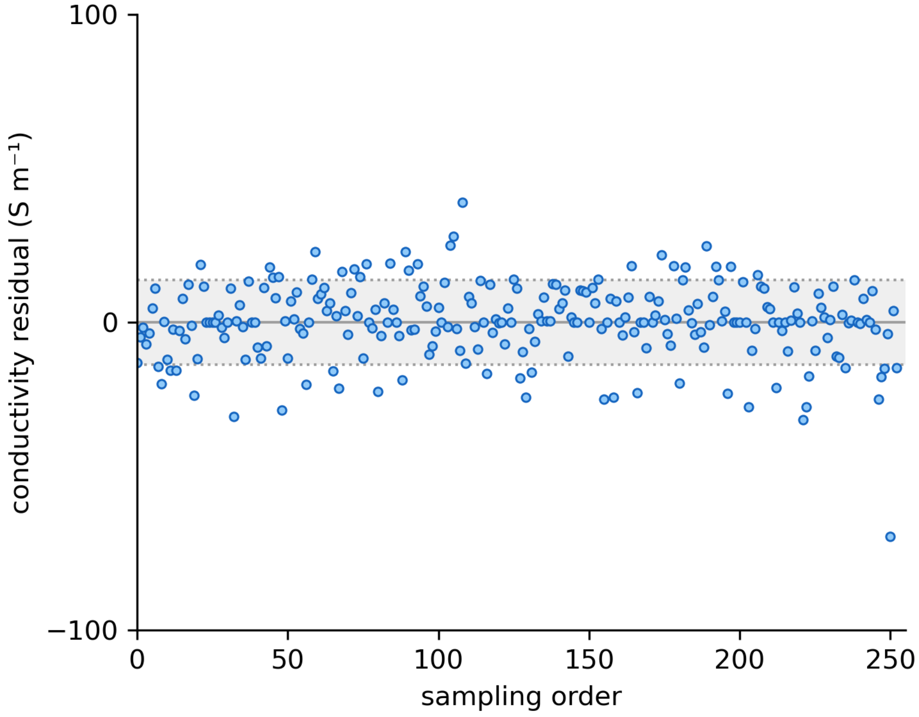

Supplementary Figure 8 | Model residuals as a function of sampling order. The conductivity residuals from the Leave-One-Out Cross-Validation (LOOCV) analysis are plotted as a function of the sampling order. The amplitude of the experimental noise in the conductivity training data was estimated using a white noise kernel in the Gaussian process regression model. This noise estimate is plotted as a grey band spanning ±1 standard deviation.

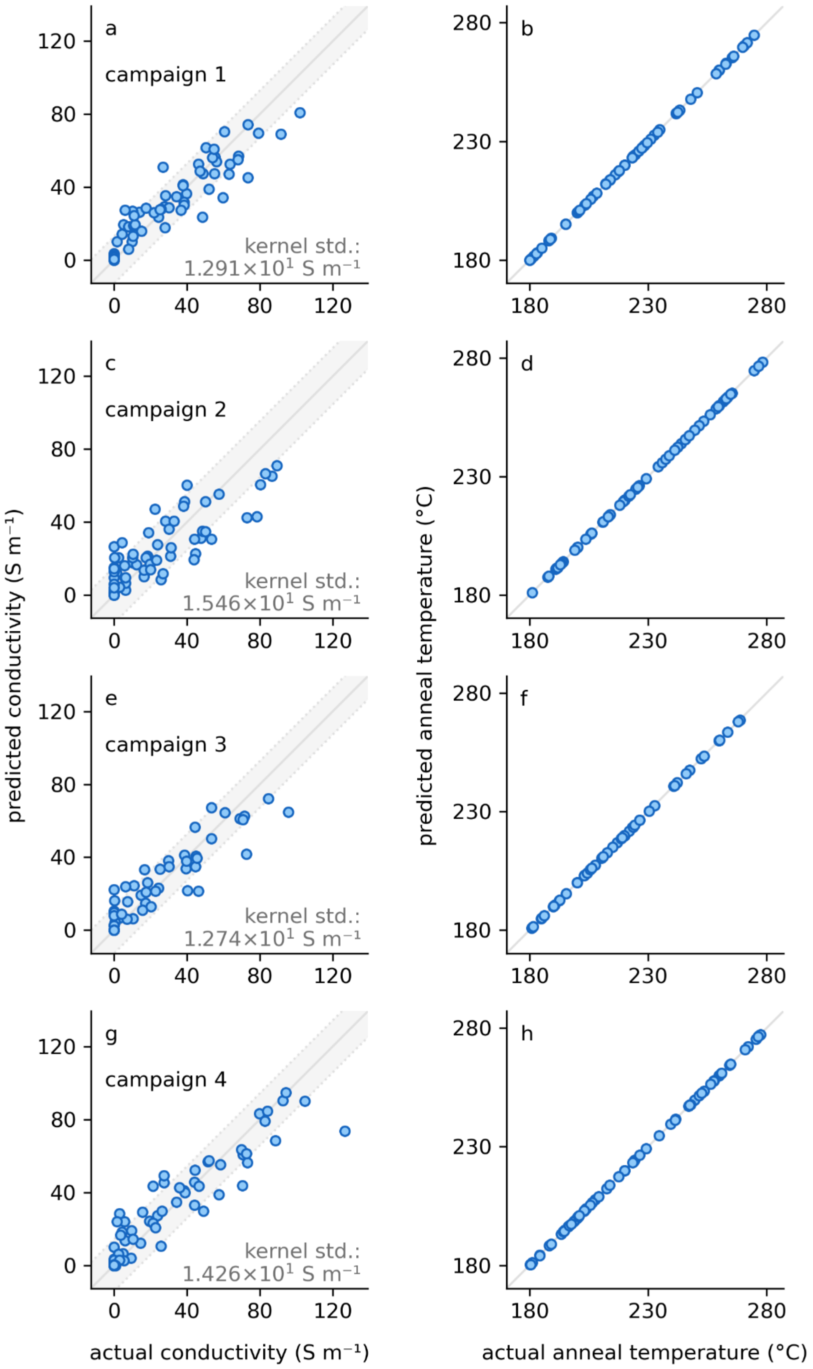

Supplementary Figure 9 | Leave-One-Out Cross-Validation (LOOCV) analysis of each of the four experimental campaigns. Cross-validation plots for the conductivity and temperature models are shown for campaign 1 (**a**,**b**), campaign 2 (**c**,**d**), campaign 3 (**e**,**f**) and campaign 4 (**g**,**h**). The amplitude of the experimental noise in the conductivity training data was estimated using a white noise kernel in the Gaussian process regression model. The RMSE and noise estimate of the conductivity model was calculated for each experimental campaign. This noise estimate is plotted as a grey band spanning ±1 standard deviation (a, c, e, and g).

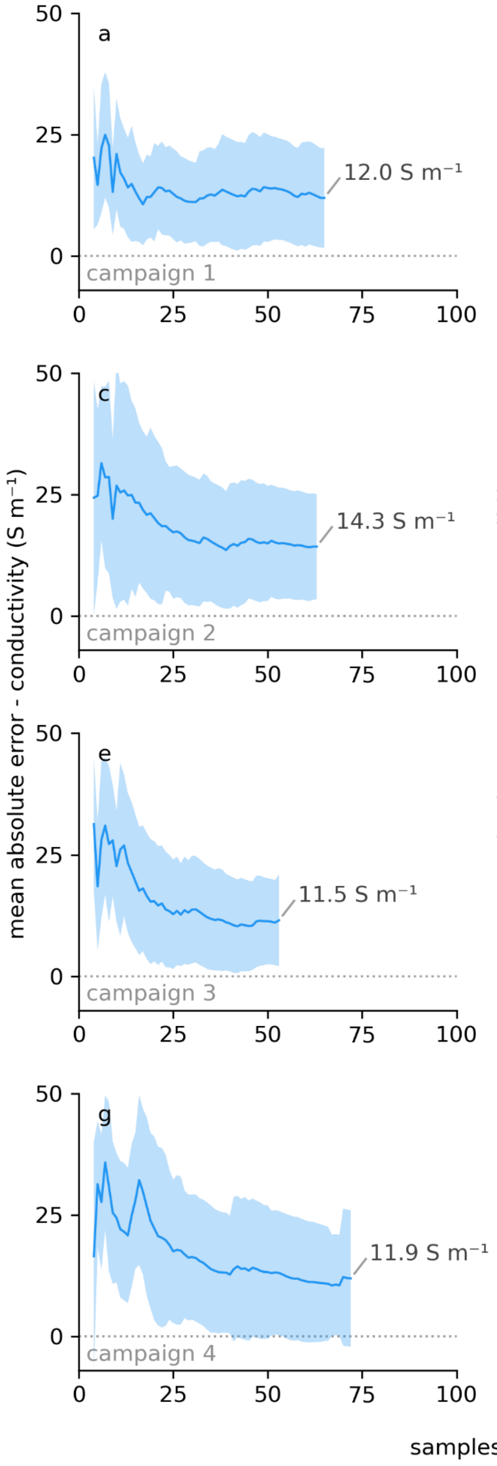

Supplementary Figure 10 | Leave-One-Out Cross-Validation (LOOCV) analysis of each of the four experimental campaigns at each sample. Cross-validation plots for the conductivity and temperature models are shown for campaign 1 (**a**,**b**), campaign 2 (**c**,**d**), campaign 3 (**e**,**f**) and campaign 4 (**g**,**h**). The LOOCV was computed after each sample was observed. The mean and standard deviation of the absolute cross-validation errors are plotted as a function of the number of samples observed.

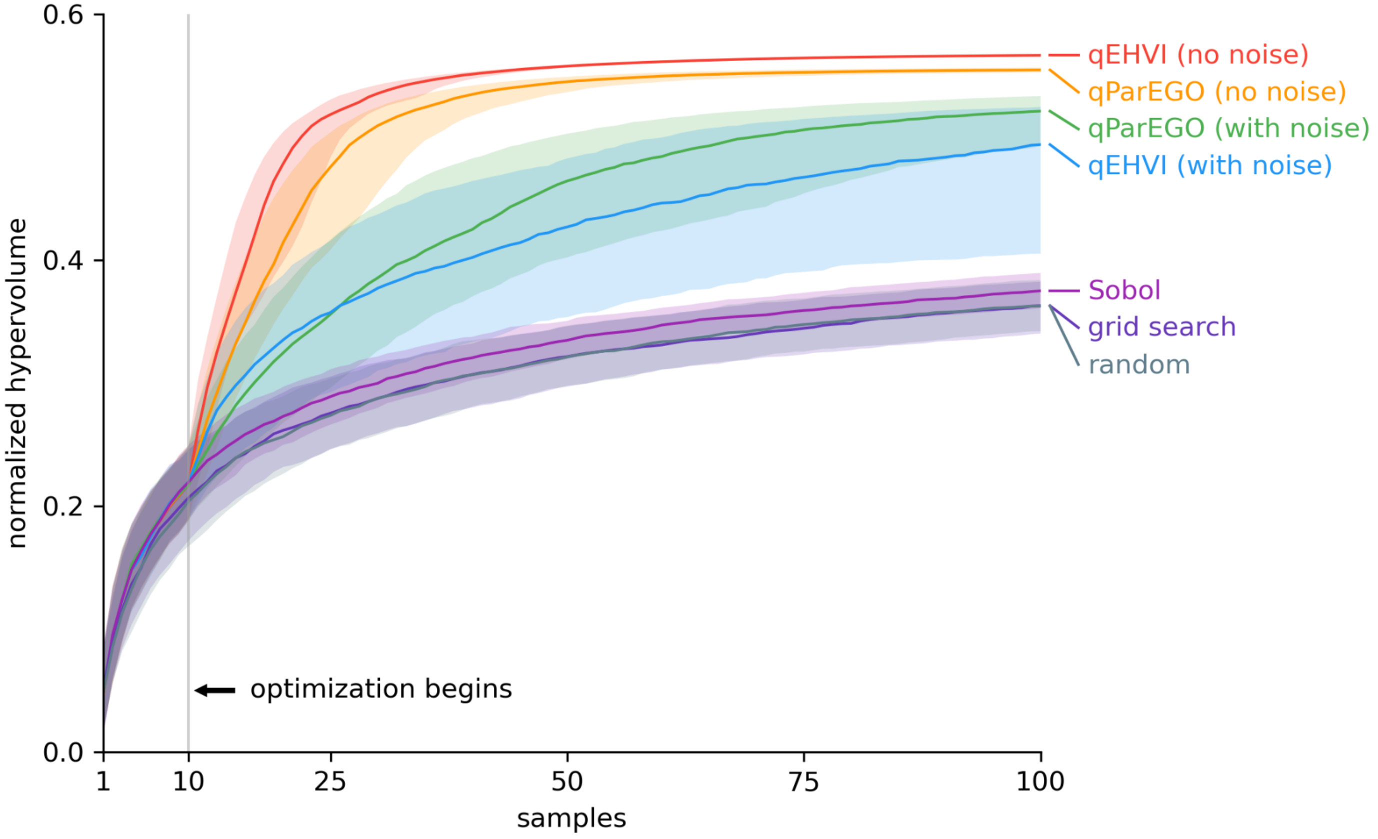

Supplementary Figure 11 | Benchmarking of qEHVI against alternative multi-objective sampling strategies. The performance of the qEHVI algorithm is compared to alternative multi-objective sampling strategies (namely the qParEGO algorithm, grid search, random sampling, and Sobol sampling) in simulated optimization campaigns, both with and without experimental noise. The median (solid line) and interquartile range (shaded bands) from replicate simulations are shown. 1000 replicate simulations were performed for all methods except random, for which random where 100,000 replicates were performed.

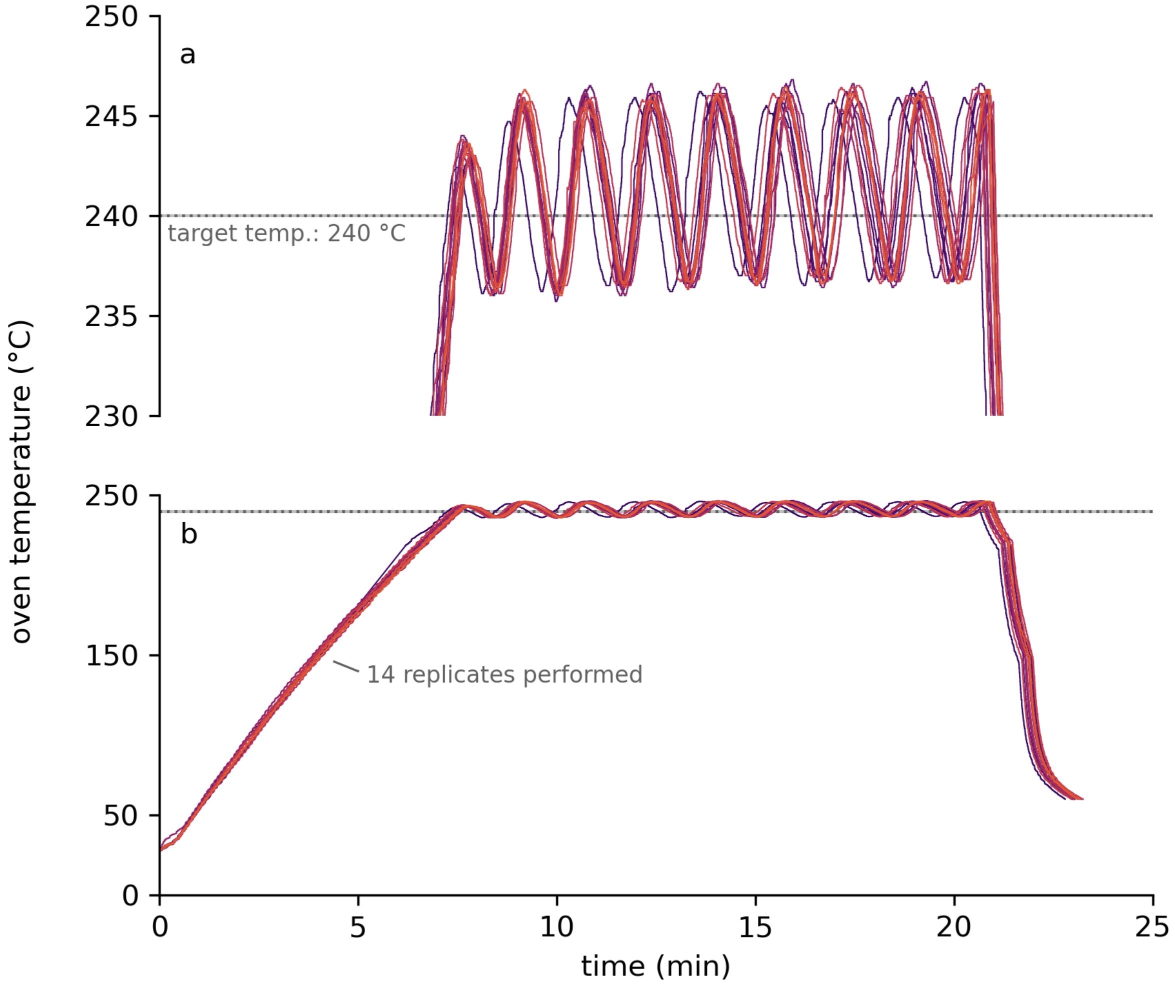

Supplementary Figure 12 | Oven temperature repeatability analysis. The temperature of the annealing oven was recorded as a function of time when the oven was set to 240 °C. This experiment was repeated 14 times. (**a**) temperature fluctuations about the setpoint (**b**) overall temperature profile.

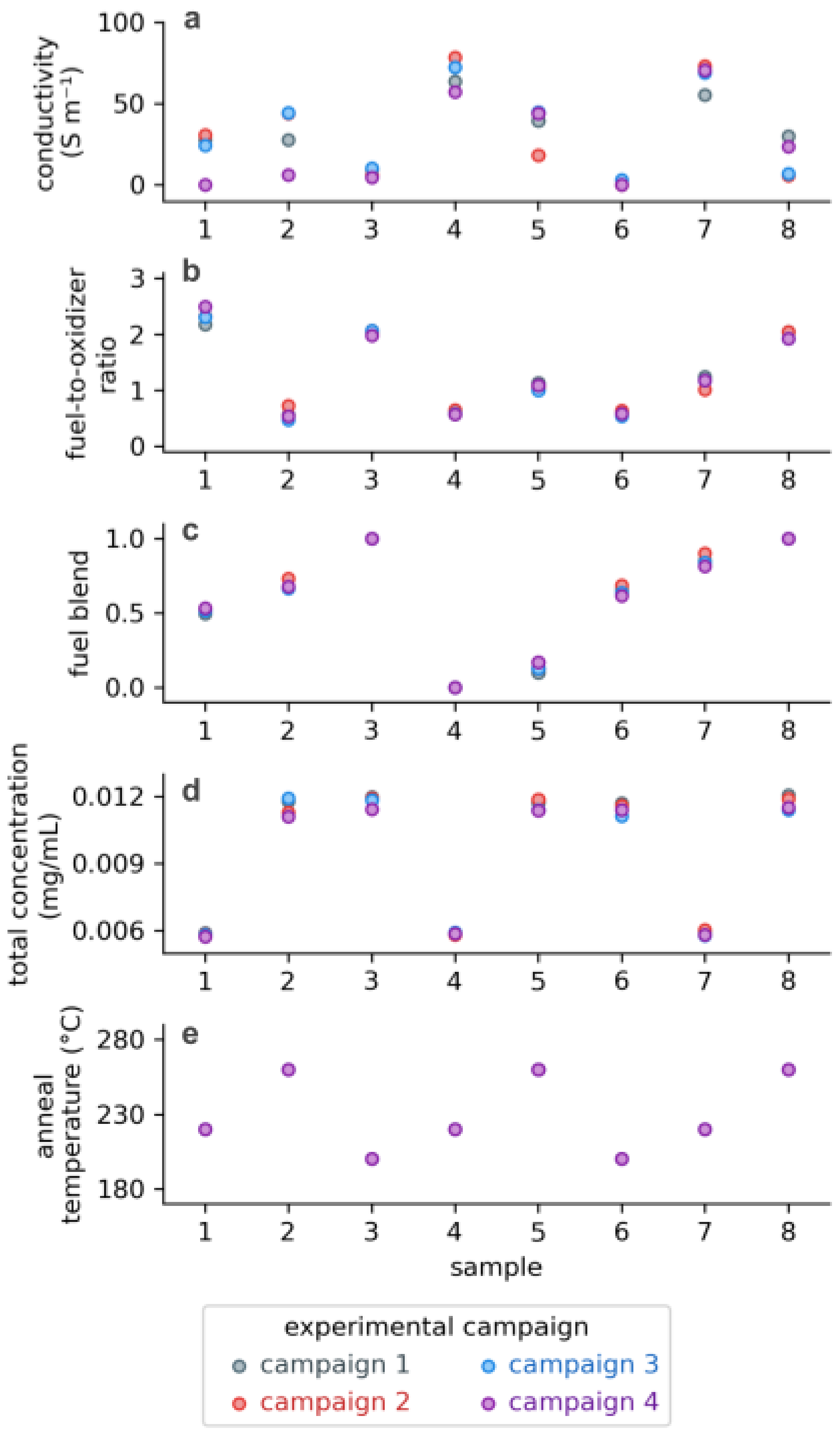

Supplementary Figure 13 | Repeatability of experimental recipe conditions and conductivity measurements. The first eight experiments of each of the four campaigns have the same targeted experimental conditions. Variations in dispensed precursor volumes cause variations in the realized experimental conditions. (**a**) measured conductivity (**b**) experimentally realized fuel:oxidizer ratio (**c**) experimentally realized fuel blend (**d**) experimentally realized total concentration (**e**) annealing temperature setpoint

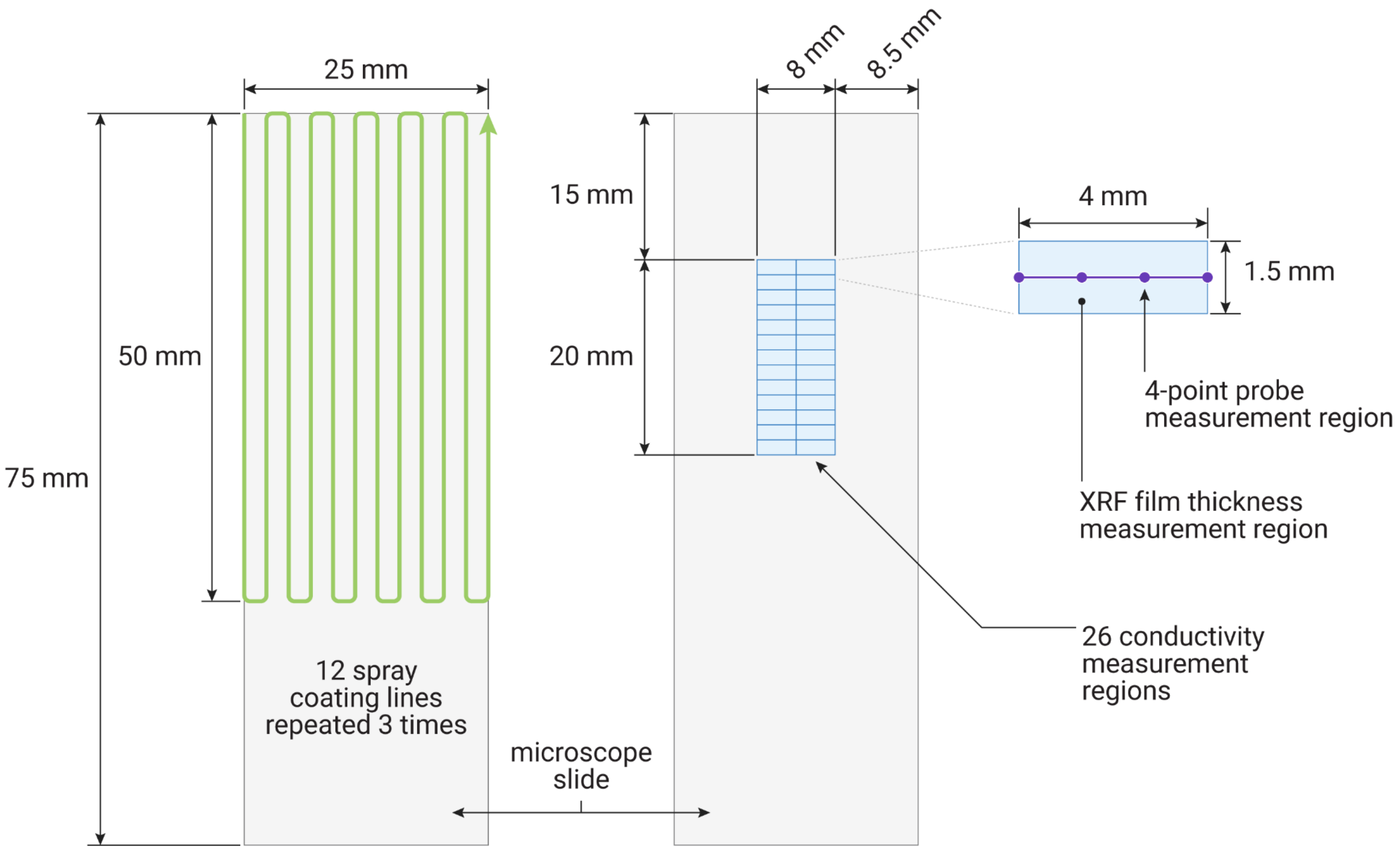

Supplementary Figure 14 | Spray coating pattern and conductivity measurement region. Four-point probe conductance and XRF thickness measurements were performed within the measurement region to calculate a conductivity value for the film.

Supplementary References

1. Jain, S. R., Adiga, K. C. & Pai Verneker, V. R. A new approach to thermochemical calculations of condensed fuel-oxidizer mixtures. *Combust. Flame* **40**, 71–79 (1981).

We gratefully acknowledge the efforts of the contributors to the many open-source software packages used in this work:

- python (https://www.python.org/)
- NumPy (https://numpy.org/)
- pandas (https://pandas.pydata.org/)
- Matplotlib (https://matplotlib.org/)
- SciPy (https://www.scipy.org/)
- scikit-learn (https://scikit-learn.org/)
- Keras (https://keras.io/)
- TensorFlow (https://www.tensorflow.org/)
- Plotly (https://plotly.com/)
- LMFIT (https://lmfit.github.io/lmfit-py/)
- HyperSpy (https://hyperspy.org/)
- luigi (https://github.com/spotify/luigi)
- Ax (https://ax.dev/)
- BoTorch (https://botorch.org/)
- PyTorch (https://pytorch.org/)
- Flask (https://flask.palletsprojects.com/)
- OBS Studio (https://obsproject.com/)
- Git (https://git-scm.com/)
